# Linking the nature and functions of sleep: insights from multimodal imaging of the sleeping brain

**DOI:** 10.1016/j.cophys.2019.11.012

**Published:** 2020-06

**Authors:** Chen Song, Enzo Tagliazucchi

**Affiliations:** 1 Cardiff University Brain Research Imaging Centre, School of Psychology, Cardiff University, Cardiff, UK; 2 Buenos Aires Physics Institute and Physics Department, University of Buenos Aires, Buenos Aires, Argentina; 3 National Scientific and Technical Research Council, Buenos Aires, Argentina

## Abstract

Sleep and wakefulness are traditionally considered as two mutually exclusive states with contrasting behavioural manifestations and complementary neurobiological functions. However, the discoveries of local sleep in global wakefulness and local wakefulness in global sleep have challenged this classical view and raised questions about the nature and functions of sleep. Here, we review the contributions from recent multimodal imaging studies of human sleep towards understanding the relationship between the nature and functions of sleep. Through simultaneous tracking of brain state and mapping of brain activity, these studies revealed that the sleeping brain can carry out covert cognitive processing that was thought to be wake-specific (wake-like function in the sleeping brain). Conversely, the awake brain can perform housekeeping functions through local sleep of neural populations (sleep-like function in the awake brain). We discuss how the blurred boundary between sleep and wakefulness highlights the need to radically rethink the definition of brain states, and how the recently discovered fMRI signatures of global and local sleep can help to address these outstanding questions.

**Current Opinion in Physiology** 2020, **15**:29–36

This review comes from a themed issue on **Physiology of sleep**

Edited by **Vladyslav Vyazovskiy** and **Jenny Morton**

For a complete overview see the Issue and the Editorial

Available online 5th December 2019

**https://doi.org/10.1016/j.cophys.2019.11.012**

2468-8673/© 2019 The Author(s). Published by Elsevier Ltd. This is an open access article under the CC BY license (http://creativecommons.org/licenses/by/4.0/).

## Introduction

Sleep is a state of behavioural quiescence, characterized by sensory-motor disconnection from the environment, and reduced levels of responsiveness and consciousness [1,2^•^]. For decades it was assumed that the brain was quiescent during dreamless sleep. This assumption, held by many eminent scientists including Charles Sherrington and Ivan Pavlov [3], was overturned by the introduction of non-invasive brain imaging techniques. It was discovered that the level of brain metabolism is similar between wake and rapid eye movement (REM) sleep and reduced by only 20% from wake to non-rapid eye movement (NREM) sleep [3,4], suggesting that the sleeping brain is highly active. The dichotomy between behaviour (quiescent) the brain (active) during sleep raises an intriguing question: are the functions of sleep causally related to this dichotomy, or is their relation epiphenomenal?

To study the relationship between the nature and functions of human sleep, a technical challenge lies in the simultaneous tracking of global brain state and recording of local brain activity. Brain activity during sleep is traditionally measured using electroencephalography (EEG), which reflects the summed postsynaptic activities of pyramidal neuron populations [5]. Although EEG has good temporal resolution and can detect rapid changes in global brain state, its spatial resolution is limited. Confounded by volume conduction, it is difficult to localize the brain regions that generate the scalp EEG signal, where different source configurations can give rise to the same EEG topography [6]. Moreover, EEG is largely insensitive to neural activity in cortical and subcortical regions far away from the scalp. By contrast, functional magnetic resonance imaging (fMRI) provides the fine spatial resolution and whole brain coverage required for recording local brain activity. At current, fMRI can already resolve signal from individual cortical columns [7] or cortical layers [8], and with advanced molecular probes, in near future fMRI will allow even higher spatial specificity, targeting individual cell types [9]. However, fMRI has limited temporal resolution. FMRI blood-oxygen-level-dependent (BOLD) signal reflects localized changes in brain blood flow and blood oxygenation, which are driven by neural activity through neurovascular coupling [10]. Because hemodynamic activities have slower response time than neural activities, fMRI cannot detect neural oscillation beyond the frequency of hemodynamic responses [11]. Given the constraints of individual brain imaging techniques, a number of studies have applied a multimodal imaging approach, such as combined EEG-fMRI, to simultaneously track global brain state and record local brain activity during human sleep [12].

In this article, we will review the contributions from recent multimodal imaging studies of human sleep towards understanding the relationship between the nature and functions of sleep. We will discuss the nature of brain processing during sleep, how it leads to reduced levels of behavioural responsiveness and consciousness on the one hand, and underlies the functions of sleep on the other hand.

## Brain signatures of global and local sleep

Traditionally, sleep has been considered to be a global state, regulated by the subcortical system and affecting the whole brain uniformly and simultaneously [13, 14, 15]. At the same time, sleep and wakefulness are treated as two mutually exclusive states, and human sleep is further divided into four stages: NREM stages N1, N2, N3 and REM [16]. N1 sleep is a transitional stage characterized by slow rolling eye movements, decreased muscle tone, and low-amplitude, high-frequency EEG activity in 4–7 Hz range (theta activity). N2 sleep is an intermediate stage characterized by increased arousal threshold and waxing-waning EEG activity in 11–16 Hz range (spindles). N3 sleep is the deepest sleep stage characterized by highest arousal threshold and high-amplitude, low-frequency EEG activity in 0.5–4 Hz range (slow waves). REM sleep is a unique sleep stage characterized by frequent dream reports, rapid eye movements, lowest muscle tone and wake-like EEG activity. In contrast to humans, sleep in rodents (the most investigated animal model) is usually divided into only two stages: NREM and REM [17].

This classical view of sleep as a global state has been challenged by the discoveries of local sleep in global wakefulness, and local wakefulness in global sleep [13, 14, 15]. When an individual is awake, single neurons or single brain regions can display brief periods of sleep-like activity, accompanied by transient behavioural impairments [18^••^,^19^]. Conversely, when an individual is in deep NREM sleep, single brain regions can display wake-like activity, accompanied by conscious experiences related to the local activation [20^•^,^21^].

**Figure 1:**
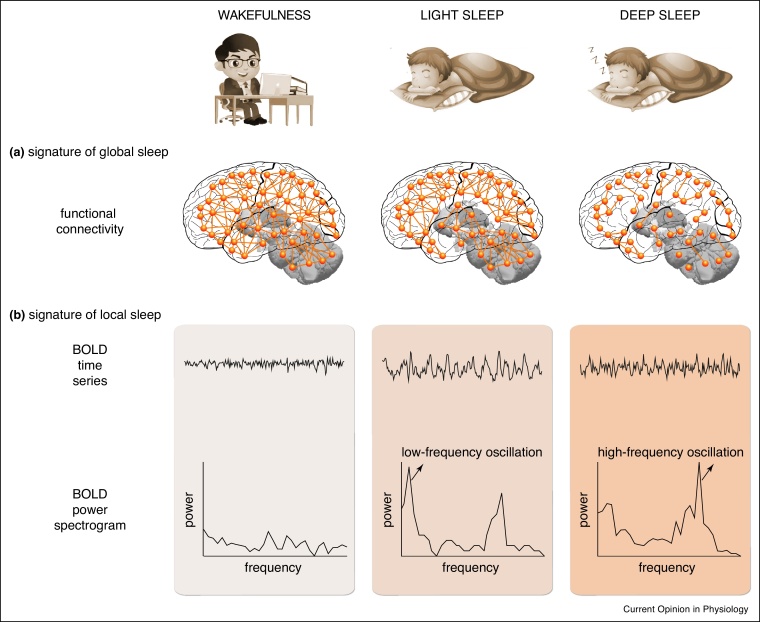
FMRI signatures of global and local sleep. Schematic illustration of the changes in global functional connectivity and local BOLD oscillation patterns from wakefulness to sleep. **(a)** Functional connectivity patterns provide a signature of global sleep, where the progression of sleep is accompanied by the gradual loss of functional connectivity, first between subcortex and cortex (wakefulness to light sleep), and then between different regions within cortex or subcortex (light sleep to deep sleep). **(b)** BOLD oscillation patterns provide a potential signature of local sleep, where the BOLD activity of individual neural populations individual fMRI voxels evolves from a mixed-frequency pattern in wakefulness, to a low-frequency (∼0.05 Hz) oscillation prominent in light sleep, and a high-frequency (∼0.17 Hz) oscillation prominent in deep sleep. The low-frequency and high-frequency BOLD oscillations track the occurrences of sleep spindles and slow waves, respectively.

It is now widely accepted that sleep and wakefulness can both occur and be regulated at a local level [13, 14, 15]. However, our understanding of local sleep and local wakefulness is still very limited. Multimodal imaging may shed new light on this by allowing the simultaneous tracking of global and local brain states. Using simultaneous EEG-fMRI, different stages of global sleep were found to be associated with distinct patterns of inter-regional interactions (Figure 1a) [22,23,24^••^], characterized as functional connectivity (correlation between the activities of two brain regions) or effective connectivity (causal influence one brain region exerts upon another) [25]. Overall, the progression from wakefulness to deep sleep is accompanied by the gradual breakdown of inter-regional interactions [22,23,24^••^]. The lightest stage of NREM sleep (N1) uncouples the thalamus and hypothalamus from the cortex, while preserving the functional interactions between cortical regions [24^••^]. The deeper stage of NREM sleep (N2) brings the loss of cortico–cortical interactions, and the deepest stage of NREM sleep (N3) further reduces the functional interactions between cortical regions belonging to different modules [24^••^]. These empirical findings are consistent with leading theories on sleep and consciousness, where the breakdown of thalamocortical interactions is thought to underlie the reduced level of behavioural responsiveness, and the loss of cortico–cortical interactions the reduced level of consciousness [26,27].

While the pattern of inter-regional interactions provides a signature of global sleep, the oscillation of fMRI blood-oxygen-level-dependent (BOLD) activity instead offers a potential signature of local sleep (Figure 1b) [28,29,30^••^]. During the transition from wakefulness to deep sleep, BOLD activity evolves from a mixed-frequency pattern to one dominated by two distinct oscillations: a low-frequency (∼0.05 Hz) oscillation that is prominent in light NREM sleep and correlates with the occurrence of sleep spindles, and a high-frequency oscillation (∼0.17 Hz) that is prominent in deep NREM sleep and correlates with the occurrence of sleep slow waves [30^••^]. The spontaneous BOLD oscillations are detectable across the whole brain, cortically and subcortically [30^••^]. They occur at the level of individual neural populations (individual fMRI voxels) [30^••^] and may reflect the intensity of local sleep.

Together these findings suggest that fMRI can track both the global brain state, by probing the functional interactions between different brain regions [22,23,24^••^], and the local state, by probing the intrinsic oscillations of individual neural populations [28,29,30^••^]. These fMRI signatures may be employed to study the relationship between global and local sleep, such as whether the functions of local sleep resemble or complement these of global sleep.

## Sleep to rewire and optimize brain circuitry

Sleep serves a number of important functions, including most prominently, the restoration of performance, conservation of energy, and consolidation of learning and memory [1,2^•^,^3^]. These seemingly different functions of sleep can be unified under the synaptic homeostasis hypothesis [2^•^]. According to this theory, while awake, the brain has to constantly learn and adapt to the environment, which involves the strengthening of synaptic connections. As a drawback, the capacity for synaptic potentiation saturates, the cost of metabolic energy increases, and the signal-to-noise-ratio of brain processing decreases. During sleep, the sensory-motor disconnection from the environment facilitates the downscaling of synaptic connections. Homeostatic downscaling effectively prunes the weak and noisy connections, while stabilizing the stronger ones, thereby consolidating learning and memory, improving the energy efficacy of brain activity, and restoring the signal-to-noise-ratio of brain processing. This theory is supported by genetic, molecular, cellular and system-level studies in humans and animals, which revealed nearly twenty percent synaptic downscaling after sleep [31, 32, 33, 34, 35].

Alternative theories, such as the trace reactivation hypothesis and the system consolidation hypothesis, propose that during sleep, the spontaneous reactivation of recently formed memory traces leads to the strengthening of synaptic connections and the consolidation of learning and memory [36,37]. These theories have also received empirical supports in humans and animals [38]. Notably, the two proposals, downscaling versus strengthening of synaptic connections, are not necessarily contradictory but may instead manifest independently and selectively in different subsets of synapses. This possibility was recently tested, where simultaneous EEG-fMRI was applied to track the dynamic change in brain state and the dynamic reorganization of brain circuitry [39^••^,^40^,^41^].

**Figure 2:**
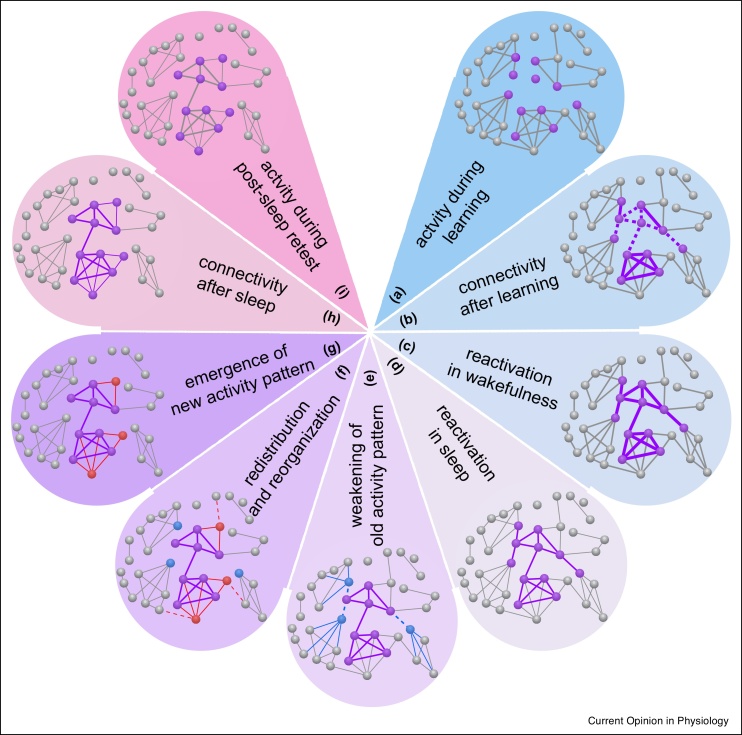
Sleep to rewire and optimize brain circuitry. Schematic illustration of the functional role that synaptic downscaling and strengthening during sleep play in memory consolidation. Each dot represents a neural population; each line represents a neural connection, where the thickness of the line indicates the strength of the connection. **(a**–**b)** Directly after learning a new task, increased functional connectivity is observed between neural populations activated during learning (marked in purple). This includes the strengthening of existing connections (solid lines) and the formation of new connections (dotted lines). **(c**–**d)** Increased connectivity facilitates the spontaneous reactivation of the same neural populations in post-learning wakefulness and sleep. However, the reactivation gets progressively weaker over the course of sleep, as the functional connectivity between the reactivated neural populations (purple lines) is downscaled to pre-learning baseline, together with a global downscaling of functional connectivity across the brain (grey lines). **(e**–**g)** Moreover, the reactivation is gradually replaced by a new activity pattern, through the reduced involvement of neural populations that are less connected to the reactivated than to other neural populations (marked in blue), and the new involvement of neural populations that are more connected to the reactivated than to other neural populations (marked in red). The reorganization of connectivity effectively pulls neural populations in the new activity pattern together (solid lines) and pushes them away from other neural populations (dotted lines). **(h**–**i)** The new activity pattern dominates in the post-sleep retest and the new connectivity pattern forms the basis of long-term storage.

Synaptic downscaling and strengthening were found to jointly underlie the consolidation of transient memory traces into long-term storage. Directly after learning a new task, increased functional connectivity is observed between neural populations activated during the learning (Figure 2a–b) [39^••^,^40^,^41^]. The increased functional connectivity facilitates the spontaneous reactivation of the same neural populations in the post-learning wakefulness and sleep (Figure 2c–d) [39^••^,^40^, ^41^, ^42^, ^43^, ^44^]. However, the reactivation gets progressively weaker over the course of sleep, as the functional connectivity between the reactivated neural populations is downscaled to pre-learning baseline [39^••^], together with a global downscaling of functional connectivity across the brain [22,23,24^••^]. Moreover, the reactivation is gradually replaced by a new activity pattern (Figure 2e–g), whose functional connectivity increases over the course of sleep, dominates in the post-sleep retest, and predicts the overnight behavioural gains (Figure 2h–i) [39^••^]. These findings suggest that memory consolidation during sleep takes place through two complementary processes: the downscaling of transient memory traces formed during pre-sleep learning, and the reorganization of memory traces into long-term storage.

The switch in the polarity of synaptic plasticity from potentiation (wake) to downscaling (sleep) is mediated not only by the sensory-motor disconnection from the environment but also by the reduced level of neuromodulators, including norepinephrine, serotonin, histamine and hypocretin, in sleep compared to wakefulness [45,46]. These background conditions, however, are not present during local sleep in global wakefulness. As such, do local sleep and global sleep serve different functions through different mechanisms, or does local sleep in global wakefulness also serve the functions of energy conservation, performance restoration and memory consolidation in a way similar to global sleep?

While the functions of global sleep are well studied, the functions of local sleep in global wakefulness remain largely unclear [47,48]. Local sleep may be the necessary by-product of global wakefulness, where different neurons will become overtired at different time points during wakefulness, depending on their history of activation. By allowing individual neurons to have cellular maintenance [49] without losing behavioural responsiveness and consciousness at a system level, local sleep may ensure the instantaneous restoration of performance and conservation of energy. Moreover, local sleep may improve the signal-to-noise ratio of brain processing and facilitate the encoding of learning and memory, by effectively lowering the activity level, raising the threshold level, and forcing neurons to signal or encode only the most salient events. To understand the exact functions of local sleep in global wakefulness, future research may apply multimodal imaging for the simultaneous tracking of local brain state, brain functionality, and brain circuitry.

## Covert sensory and cognitive processing during sleep

The functions of sleep are traditionally studied with respect to the endogenous brain processing during sleep. In the past few years, there has been a paradigm shift towards exploiting the exogenous, stimulus-driven brain processing during sleep. Although the behavioural responses to sensory stimulation are largely absent in sleep, the brain can nonetheless process and respond to sensory stimulation to a certain extent [50^•^]. The dissociation between behavioural responses and brain responses provides a unique opportunity by which external sensory stimulation can be delivered to perturb sleeping brain activity [51^•^,^52^,^53^] or evoke covert cognitive processing [54,55^•^,^56^,^57^] without waking up participants. Mapping the extent of sensory processing during sleep [58, 59, 60,61,62,63] is essential for understanding how the functions of sleep can be modulated externally.

**Figure 3:**
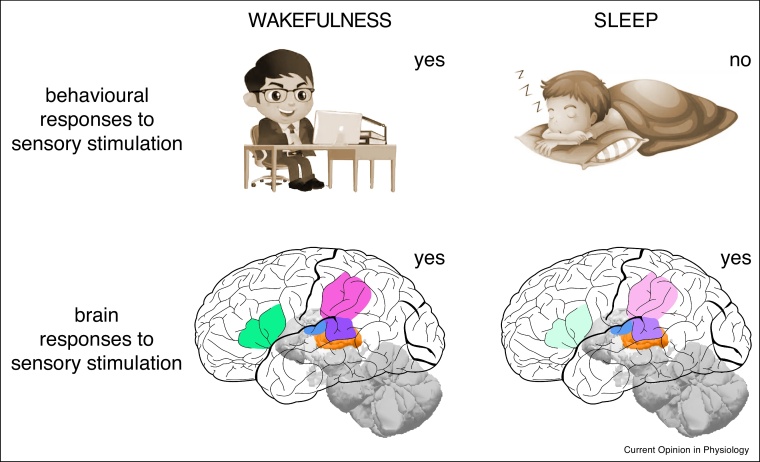
Unconscious sensory processing during sleep. Schematic illustration of the behavioural and brain responses to external sensory stimulation during sleep compared to wakefulness. While the behavioural responses to sensory stimulation are largely absent in sleep, the brain can nonetheless process and respond to sensory stimulation to a certain extent. Illustrated in the lower panel are the brain responses to auditory stimulation, where orange, blue, purple, pink, and green marks the thalamus, primary auditory cortex, Wernicke's Area, Supramarginal Gyrus, and Broca's Area, respectively, and the saturation of the colour indicates the level of processing.

Converging evidence from simultaneous EEG-fMRI studies revealed that sensory processing is preserved in thalamus but gradually reduces from thalamus to primary sensory cortices to higher cortical regions during sleep (Figure 3) [58, 59, 60,61,62,63]. Compared to wakefulness, auditory stimulation delivered during sleep evokes similar level of BOLD responses in thalamus, but significantly reduced responses in primary auditory cortex, and severely reduced or completely abolished responses in higher cortical regions [58, 59, 60,61,62,63]. The reduced responses are accompanied by a loss of feature selectivity: Wernicke’s and Broca’s areas, which respond selectively to comprehensible sentences in wakefulness, lose their ability to differentiate comprehensible and meaningless sentences during sleep [59]. The gradual diminishing of sensory processing along cortical hierarchy is likely to result from the breakdown of thalamocortical and cortico–cortical interactions [22,23,24^••^].

Importantly, sensory processing during sleep is non-stationary and depends heavily on endogenous brain activity [61,62,63]. Auditory stimulation can evoke BOLD responses in thalamus and primary auditory cortices during the majority of NREM sleep, expect when the stimulation is delivered during spindles or during the downstate of slow waves, where the evoked BOLD responses become less consistent or even absent [61,62]. In REM sleep, auditory stimulation can evoke BOLD responses in thalamus and primary auditory cortices if the level of rapid eye movement is low (tonic REM sleep), but not if the level is high (phasic REM sleep) [63].

The dependence of sensory processing on spontaneous brain activity points towards the existence of optimal time windows during which external sensory stimulation can affect the sleeping brain most effectively. Specifically, the upstate of slow waves may represent an optimal time window, as it indicates periods of increased neural excitability [2^•^]. In line with this hypothesis, auditory stimulation delivered during the upstate of slow waves was shown to be highly effective, for creating new semantic associations [56], reactivating existing memory [57], enhancing subsequent slow wave activity [51^•^,^52^] and boosting the immune system [53]. By contrast, auditory stimulation delivered during the downstate of slow waves fails to evoke covert cognitive processing [57] and has disruptive effects on the following slow wave activity [51^•^,^52^].

These studies suggest that the sleeping brain is capable of carrying out covert cognitive processing that was thought to be wake-specific. This includes encoding external sensory stimulation [54], preparing for motor responses [55^•^], building semantic associations [56], reactivating memory [57], and possibly more functions yet to be discovered. A future challenge is to map the boundaries of covert cognitive processing during sleep and to study, at a mechanistic level, the similarity or difference to cognitive processing during wakefulness.

## Conclusions and future directions

Our understanding of sleep has been reshaped by multimodal imaging studies in the past few years. Sleep and wakefulness are no longer considered to be mutually exclusive. Instead, the sleeping brain can carry out covert sensory and cognitive processing that was thought to be wake-specific. Conversely, in the awake brain, individual neural populations can undertake brief periods of local sleep for cellular maintenance, performance restoration and energy conservation.

The simultaneous occurrence of sleep and wakefulness, such as local sleep in global wakefulness, or local wakefulness in global sleep, highlights the need to rethink the definition of brain states. Given the interconnectedness of the brain, the functions of local neural populations can be influenced not only by their own state but also by the global brain state. When the local and global states conflict, such as during local sleep in global wakefulness, or local wakefulness in global sleep, do the functions of local neuronal populations reflect more their own state or the global brain state? Moreover, shall local sleep in global wakefulness and local wakefulness in global sleep be considered as singular states that differ from global sleep and global wakefulness in their nature as well as functions? Future research may employ the fMRI signatures of global and local brain states to address these outstanding questions.

## Conflict of interest statement

Nothing declared.

## References and recommended reading

Papers of particular interest, published within the period of review, have been highlighted as:

• of special interest
•• of outstanding interest
